# Negative Regulation of Notch Signaling by Xylose

**DOI:** 10.1371/journal.pgen.1003547

**Published:** 2013-06-06

**Authors:** Tom V. Lee, Maya K. Sethi, Jessica Leonardi, Nadia A. Rana, Falk F. R. Buettner, Robert S. Haltiwanger, Hans Bakker, Hamed Jafar-Nejad

**Affiliations:** 1 Department of Molecular and Human Genetics, Baylor College of Medicine, Houston, Texas, United States of America; 2 Department of Cellular Chemistry, Hannover Medical School, Hannover, Germany; 3 Program in Developmental Biology, Baylor College of Medicine, Houston, Texas, United States of America; 4 Department of Biochemistry and Cell Biology, Stony Brook University, Stony Brook, New York, United States of America; Harvard Medical School, Howard Hughes Medical Institute, United States of America

## Abstract

The Notch signaling pathway controls a large number of processes during animal development and adult homeostasis. One of the conserved post-translational modifications of the Notch receptors is the addition of an *O*-linked glucose to epidermal growth factor-like (EGF) repeats with a C-X-S-X-(P/A)-C motif by Protein *O*-glucosyltransferase 1 (POGLUT1; Rumi in *Drosophila*). Genetic experiments in flies and mice, and *in vivo* structure-function analysis in flies indicate that *O*-glucose residues promote Notch signaling. The *O*-glucose residues on mammalian Notch1 and Notch2 proteins are efficiently extended by the addition of one or two xylose residues through the function of specific mammalian xylosyltransferases. However, the contribution of xylosylation to Notch signaling is not known. Here, we identify the *Drosophila* enzyme Shams responsible for the addition of xylose to *O*-glucose on EGF repeats. Surprisingly, loss- and gain-of-function experiments strongly suggest that xylose negatively regulates Notch signaling, opposite to the role played by glucose residues. Mass spectrometric analysis of *Drosophila* Notch indicates that addition of xylose to *O*-glucosylated Notch EGF repeats is limited to EGF14–20. A *Notch* transgene with mutations in the *O*-glucosylation sites of Notch EGF16–20 recapitulates the *shams* loss-of-function phenotypes, and suppresses the phenotypes caused by the overexpression of human xylosyltransferases. Antibody staining in animals with decreased Notch xylosylation indicates that xylose residues on EGF16–20 negatively regulate the surface expression of the Notch receptor. Our studies uncover a specific role for xylose in the regulation of the *Drosophila* Notch signaling, and suggest a previously unrecognized regulatory role for EGF16–20 of Notch.

## Author Summary

In multi-cellular organisms, neighboring cells need to communicate with each other to ensure proper cell fate decisions and differentiation. Signaling through the Notch receptors is the primary means by which local cell-cell communication is accomplished in animals. Given the broad usage of Notch signaling in animals and the host of human disease caused by Notch pathway misregulation, sophisticated mechanisms are required to adjust the strength of Notch signaling in each context. We have previously shown that addition of glucose residues to the Notch receptor promotes Notch signaling. Since these glucose residues on Notch can be extended by addition of xylose residues, we sought to determine whether xylose also plays a role in the regulation of Notch signaling. In contrast to glucose, we determine that xylose residues decrease Notch signaling in certain contexts by controlling Notch surface levels. Moreover, the xylose residues reside in a specific domain of Notch, unlike the glucose residues which are distributed throughout the Notch extracellular domain. Our data provide an example of signaling pathway regulation by altering the distribution of the short or elongated forms of a saccharide on a receptor protein, and offer a potential avenue for modulating Notch signaling as both a therapeutic modality and a tool in regenerative medicine.

## Introduction

Notch signaling is a juxtacrine cell-cell communication pathway with broad roles in animal development and adult tissue homeostasis [1], [2]. Both gain- and loss-of-function mutations in Notch pathway components cause human disease [3]–[5], and therapeutic approaches to alter the activity of Notch signaling are a subject of intense research and development [6]. The extracellular domains of Notch receptors contain a large number (up to 36) of EGF repeats. Each EGF repeat contains six cysteine residues, which are linked to each other via three disulfide bonds [7]. Of the several forms of *O*-linked carbohydrates found on Notch EGF repeats [8]–[10], two have been shown to be required for Notch signaling in both flies and mammals: *O*-fucose and *O*-glucose [11]–[16]. Addition of *N*-acetylglucosamine (GlcNAc) to *O*-fucose by Fringe glycosyltransferases modulates Notch signaling in several contexts [17], [18].

**Figure 1:**
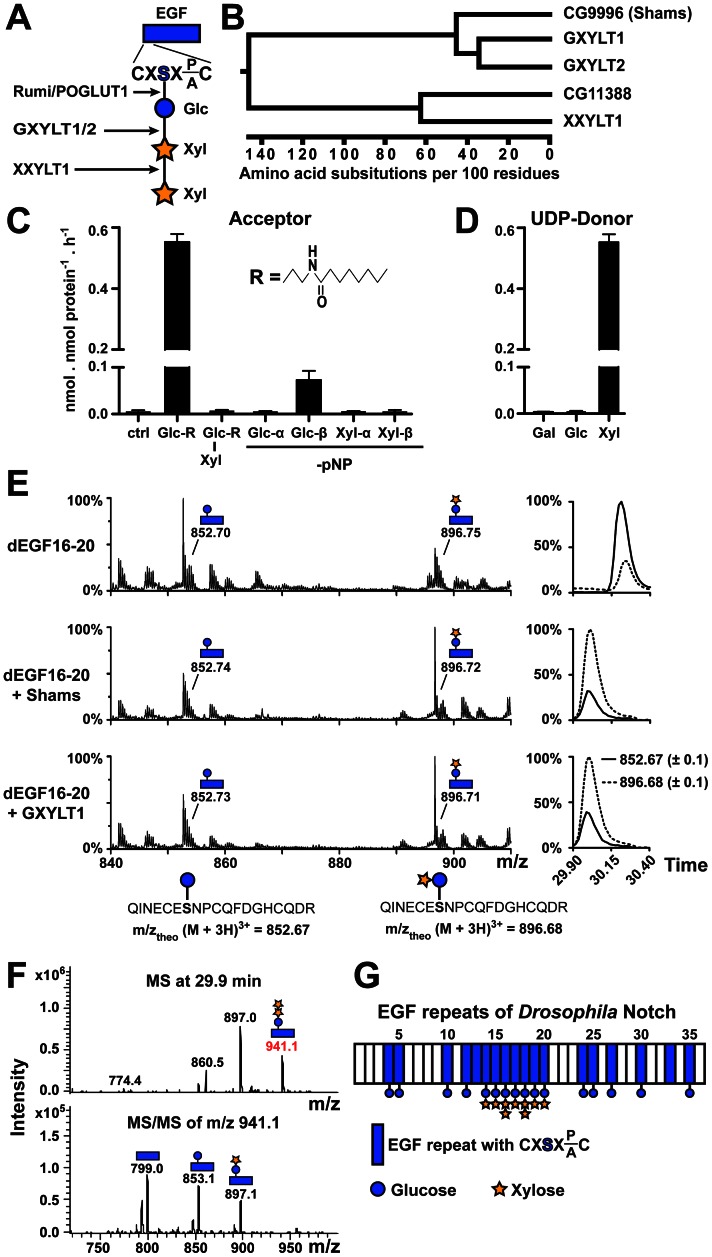
Shams functions as a glucoside xylosyltransferase on Notch. (A) Schematic of the xylose-xylose-glucose trisaccharide attached to the serine (S) residue in the consensus sequence on an EGF repeat and the glycosyltransferases involved in its generation. (B) Phylogenetic tree of human GXYLT1/2 and XXYLT1, and their *Drosophila* homologs CG9996 (Shams) and CG11388 based on the Clustal W algorithm. (C) Xylosyltransferase assays using UDP-[^14^C]xylose donor and synthetic lipophilic acceptors to determine acceptor specificity of Shams. R represents the drawn hydrophobic aglycon; pNP, *para*-nitrophenol. (D) Donor substrate specificity using Glc-R as substrate. (E) Mass spectrometric analysis of a glycosylated peptide of *Drosophila* Notch (d) EGF16–20 expressed in Sf9 insect cells shows an increase in the ratio of disaccharide- versus monosaccharide-modified form after incubation with Shams or human GXYLT1 and UDP-xylose *in vitro*. Extracted ion chromatograms on the right indicate that the ratio between xylosylated and non-xylosylated peptides (dashed and solid lines, respectively) is inverted by Shams and GXYLT1. (F) Mass spectrometry demonstrates the presence of *O*-glucose trisaccharide on a peptide from EGF16 (^639^QINECE**S**NPCQFDGHCQDR^657^). Top and bottom panels show MS and MS/MS spectra, respectively. (G) Schematic representation of sites of dNotch xylosylation identified by mass spectrometry (spectra shown in Figure S2). The most elongated glycan structure detected on each EGF repeat is shown, but the shorter forms can also exist.

*O*-linked glucose is attached to serine residues in the consensus sequence C^1^-X-S-X-(P/A)-C^2^ by the protein *O*-glucosyltransferase Rumi/POGLUT1 [8], [14], [19]. Rumi is required for both fly and mammalian Notch signaling at a step downstream of ligand-binding [14], [15], [20]. *In vivo* structure-function analyses indicate that all of the 18 Rumi target sequences in *Drosophila* Notch (dNotch) contribute to Notch activation, with *O*-glucose sites on EGF10-15 playing a more important role than others [20]. *O*-glucose can serve as a substrate for additional sugar modifications to generate xylose-xylose-glucose trisaccharides [8], [19], [21]. The human enzymes responsible for the addition of xylose to *O*-glucosylated Notch EGF repeats have recently been identified: glucoside xylosyltransferase (GXYLT)1 and GXYLT2 add the first xylose and xyloside xylosyltransferase (XXYLT1) adds the second (Figure 1A) [22], [23]. Thus far, no functional studies have been performed to analyze the role of xylosylation in Notch signaling.

Here, we identify the *Drosophila* glucoside xylosyltransferase which adds xylose to *O*-glucosylated EGF repeats and show that this enzyme negatively regulates *Drosophila* Notch signaling in certain contexts. We use a combination of mass spectrometry, genetic and *in vivo* mutational studies to show that the functionally important sites of xylosylation reside in EGF repeats 16–20 of Notch, and that xylose negatively regulates the surface expression of Notch. Given that *O*-glucose positively regulates Notch signaling in all contexts studied so far [20], negative regulation of Notch signaling by xylosylated *O*-glucose glycans provides an example of how the strength of a signaling pathway can be fine-tuned by stepwise addition of carbohydrate molecules to a receptor.

## Results

### *Drosophila CG9996* (*shams*) Encodes a Glucoside Xylosyltransferase which Adds Xylose to *O*-glucosylated Notch EGF Repeats

Using homology searches, we identified two novel *Drosophila* proteins (CG9996 and CG11388) homologous to human Notch xylosyltransferases. Sequence comparison between these two fly proteins and human Notch xylosyltransferases indicated that CG9996 is the only close fly homolog of human GXYLT1 and GXYLT2, whereas CG11388 bears much more sequence identity with XXYLT1 (Figure 1B and Figure S1). We named CG9996 Shams, a companion and muse for the poet Rumi. To test whether Shams can add xylose to glucose similar to its human homologs, we first performed *in vitro* glycosyltransferase assays by using purified, recombinant Shams and synthetic lipophilic acceptors (Figure 1C). We found that Shams can indeed add xylose specifically to a synthetic acceptor harboring a glucose residue (Glc-β1-R) but not to one harboring a xylose-glucose disaccharide (Xyl-α1,3-Glc-β1-R) (Figure 1C). To further examine the substrate specificity of Shams, we used glucose or xylose residues attached to *para*-nitrophenol (pNP) in α- or β-linkage as the acceptor for the xylosyltransferase activity of Shams and found that Shams can only transfer xylose to Glc-β1-pNP (Figure 1C). We also performed similar assays to determine the donor substrate specificity of Shams and found that Shams is able to transfer xylose, but not glucose or galactose, to the Glc-β1-R acceptor (Figure 1D). To examine whether Shams is able to add xylose to *O*-glucosylated EGF repeats of Notch, we assayed purified Shams using a fragment of *Drosophila* Notch (EGF16–20) harboring several *O*-glucosylation sites, expressed in and purified from Sf9 cells. Mass spectrometric analysis of a glycosylated peptide of EGF16 showed that Sf9 cells produce a mixture of glucose and xylose-glucose in a ratio of about 3 to 1 at this site. Sf9 cells apparently highly glucosylate EGF16, but show limited xylosylation capacity. *In vitro*, both Shams and human GXYLT1 can transfer xylose to *O*-glucosylated EGF16, changing the ratio in favor of the xylosylated form (Figure 1E). Taken together, these experiments show that Shams functions as a glucoside xylosyltransferase capable of adding the first xylose to *O*-glucose on Notch EGF repeats, similar to its human homologs GXYLT1 and GXYLT2 [23].

### Addition of Xylose to *O*-glucose Occurs on a Subset of Notch EGF Repeats in *Drosophila*

Mass spectrometry on mouse Notch1 expressed in several mammalian cell lines has shown that xylose-xylose-glucose trisaccharide is the dominant form of *O*-glucose glycans on all mouse Notch1 EGF repeats harboring an *O*-glucosylation site, although the stoichiometry varies among different EGF repeats [8], [19]. To determine the distribution of xylosylated *O*-glucose glycans on *Drosophila* Notch, we performed systematic mass spectrometric analyses of *Drosophila* Notch expressed in *Drosophila* S2 cells. We find that while *O*-glucose is found at all predicted sites analyzed, xylose is only detected on EGF14–20, and xylose-xylose-glucose trisaccharides are only detected at EGF16 and EGF18 (Figure 1F and 1G and Figure S2). Therefore, unlike mammalian Notch1, addition of xylose to *O*-glucose appears to be limited to a subset of EGF repeats of the *Drosophila* Notch.

### *shams* Mutations Promote Notch Signaling

**Figure 2:**
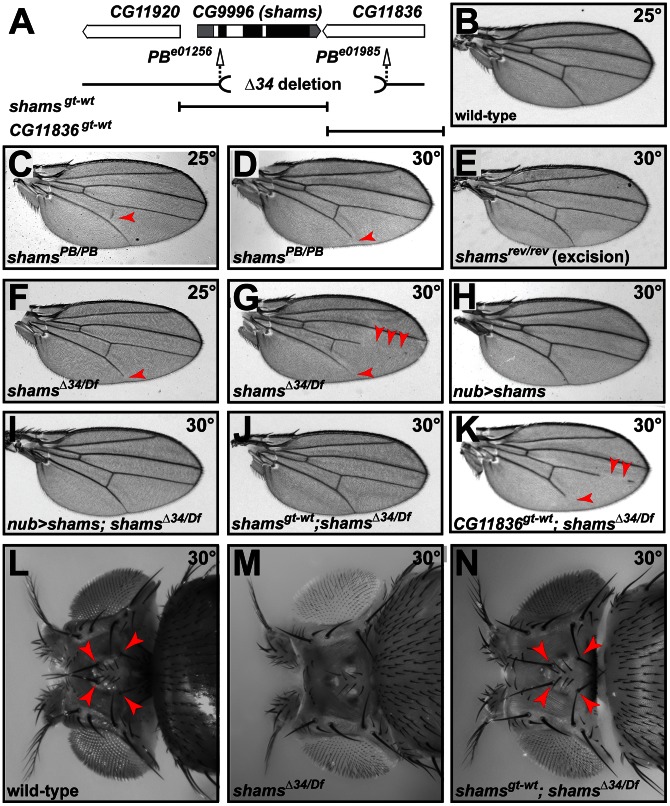
Mutations in *shams* result in the loss of wing veins and head bristles. (A) Schematic representation of the genomic region containing *shams* (*CG9996*) and its neighboring genes, *shams* alleles, and the *shams* and *CG11836* rescue transgene. Black boxes indicate the coding parts of exons. (B) Adult wing of a wild-type fly. (C,D) *shams^PB/PB^ (e01256)* mutants raised at 25°C exhibit a partially penetrant loss of the posterior cross-vein (C) and at 30°C lose the distal portion of the L5 wing vein (arrowheads) (D). (E) Precise excision of the *piggyBac* insertion results in animals with normal wing veins. (F,G) Adult wings of *shams^Δ34^/Df(3R)BSC494* flies raised at 25°C lose wing vein material at the distal end of L5 (F) and at 30°C exhibit substantial loss of L4, L5, and posterior cross-vein (G). (H,I) Overexpression of *shams* with *nubbin-GAL4* does not cause any phenotypes in the wing (H), but rescues the wing vein loss in *shams^Δ34^/Df(3R)BSC494* animals (I). (J) *shams^gt-wt^* rescues *shams^Δ34^/Df(3R)BSC494* wing defects. (K) A genomic rescue transgene harboring *CG11836* does not rescue the wing vein phenotype of *shams^Δ34^/Df(3R)BSC494* animals. (L) Wild-type adult heads have two ocellar bristles and two post-vertical bristles (arrowheads). (M) In *shams^Δ34^/Df(3R)BSC494* mutants raised at 30°C, ocellar and post-vertical bristles are lost. (N) This bristle phenotype is rescued by *shams^gt-wt^* (arrowheads).

To examine the role of xylosylation in *Drosophila* Notch signaling, we performed genetic experiments on two independent alleles of *shams* (Figure 2A). Flies homozygous for the *piggyBac* insertion *shams^e01256^* (*shams^PB/PB^*) are viable at 25°C and do not exhibit any adult phenotypes besides a loss of the posterior cross-vein in 20% of the flies (Figure 2C; compare to 2B). When raised at 30°C, 56% of *shams^PB/PB^* flies lose the distal portion of the L5 wing vein (Figure 2D), similar to the phenotype observed in gain-of-function *Abruptex* alleles of Notch (*N^Ax^*) [24]. Precise excision of this *piggyBac* insertion fully reverts the phenotype, indicating that the observed loss of the wing vein is due to the insertion (Figure 2E). We also generated a null allele lacking 97% of the Shams coding region by using FLP/FRT-mediated recombination on two *piggyBac* insertions in the region (Figure 2A) [25]. Animals homozygous or hemizygous for the null allele *shams^Δ34^* survive to adulthood and exhibit a 100% penetrant loss of the L5 wing vein at 25°C (Figure 2F). At 30°C, *shams^Δ34/Df^* animals are semi-lethal and all the escapers exhibit partial loss of multiple wing veins (Figure 2G). The wing vein loss phenotype can be rescued by overexpression of *shams* cDNA (Figure 2H and 2I), by providing a *shams^gt-wt^* genomic transgene that contains the *shams* locus (Figure 2J), or by an HA-tagged version of the *shams* genomic transgene (*shams^gt-wt-HA^*, data not shown). However, even though the *shams^Δ34^* allele also affects *CG11836* (Figure 2A), a genomic transgene containing this gene (*CG11836^gt-wt^*) does not rescue the wing vein phenotypes of the *shams^Δ34/Df^* animals (Figure 2K). Of note, each of the *shams^gt-wt^* and *CG11836^gt-wt^* genomic transgenes partially rescues the semi-lethality of these animals, indicating that both transgenes are functional. These observations indicate that loss of *shams* results in a wing vein loss phenotype. *shams^Δ34/Df^* animals raised at 30°C also lose the ocellar and postvertical bristles in the head (Figure 2M; compare to 2L) similar to *N^Ax^* alleles [26]. Again, this phenotype can be fully suppressed by a *shams* genomic transgene (Figure 2N). Together, these data indicate that loss of *shams* results in phenotypes reminiscent of *Notch* gain-of-function phenotypes.

**Figure 3:**
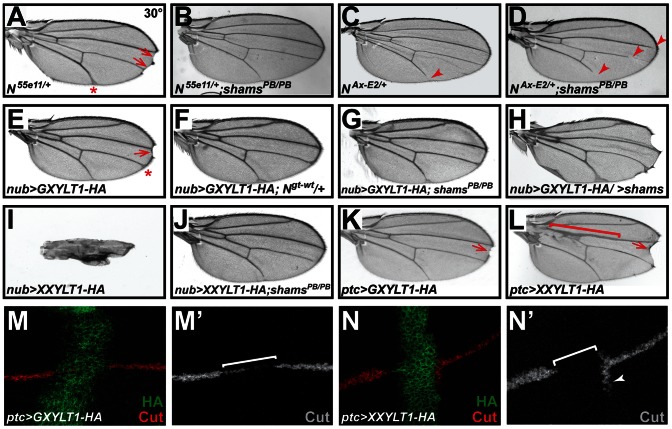
Xylosyltransferases inhibit *Drosophila* Notch signaling. All animals were raised at 30°C. (A) *N^55e11/+^* animals show wing vein thickening (asterisk) and margin defects (arrows). (B) *shams^PB/PB^* suppresses the *N^55e11/+^* phenotypes. (C) The *Abruptex* mutant *N^Ax-E2/+^* exhibits loss of wing vein at distal L5 (arrowhead) and occasionally L2. (D) *shams^PB/PB^* enhances the *N^Ax-E2/+^* phenotype. (E) Wing-specific overexpression of HA-tagged human GXYLT1 by *nubbin-GAL4* (*nub>GXYLT1-HA*) induces wing vein thickening (asterisk) and wing margin scalloping (arrow). (F) An additional copy of *Notch* suppresses the wing margin defect in *nub>GXYLT1-HA*. (G) The wing margin defect of *nub>GXYLT1-HA* flies is suppressed in a *shams^PB/PB^* background. Note that GXYLT1-HA rescues the L5 wing vein loss phenotype of *shams^PB/PB^* (compare to Figure 2D). (H) Co-overexpression of GXYLT1-HA and Shams results in the enhancement of the wing margin loss and wing vein expansion. (I) Overexpression of human *XXYLT1-HA* in the wing results in severe wing vein expansion and a complete loss of wing margin. (J) *shams^PB/PB^* fully suppresses these phenotypes, but XXYLT1-HA overexpression does not suppress the L5 vein loss phenotype of *shams ^PB/PB^*, indicating that the XXYLT1-HA phenotypes are strictly mediated by its enzymatic activity. (K) Overexpression of GXYLT1-HA by *patched-GAL4* results in a mild wing margin loss in the patched domain (arrow). (L) Overexpression of XXYLT1-HA by *patched-GAL4* results in wing vein thickening (bracket) and a more pronounced wing margin loss (arrow) in the patched domain. (M–N′) Double staining of wing imaginal discs by antibodies against HA (green) and the Notch downstream target Cut (red in M,N; gray in M′,N′). Note that the loss of Cut is less severe upon GXYLT1-HA overexpression (M,M′) compared to that resulting from XXYLT1-HA overexpression (N,N′).

To examine whether the observed phenotypes are indeed due to increased Notch signaling, we performed genetic interaction studies. As reported previously, loss of one copy of *Notch* results in wing margin defects and wing vein expansion (Figure 3A) [27]. Homozygosity for the *shams^PB^* allele suppresses the *N^55e11/+^* haploinsufficient phenotypes (Figure 3B and Figure S3). In a reciprocal experiment, we find that *shams^PB^* enhances the wing vein loss observed in the *N^Ax-E2^* dominant gain-of-function allele (Figure 3C and 3D). Together, these observations indicate that Shams decreases the activity of Notch, potentially by adding xylose to *O*-glucose residues on Notch EGF repeats.

### Overexpression of Human Xylosyltransferases Inhibits Notch Signaling

To examine the effects of increased xylosylation on Notch signaling, we overexpressed an HA-tagged version of human GXYLT1 in developing fly wings. We observed margin scalloping in 40% of adult wings at 30°C (Figure 3E) and a mild wing vein expansion at 25°C and 30°C (Figure 3E and Figure S4). Increasing the *Notch* gene dosage suppressed the wing margin loss caused by GXYLT1-HA overexpression (Figure 3F), indicating that the phenotype is due to decreased Notch signaling. Overexpression of GXYLT1-HA in a *shams^PB/PB^* background resulted in the mutual suppression of the wing scalloping and vein loss phenotypes observed in each genotype (Figure 3G; compare to Figure 3E and Figure 2D). Simultaneous overexpression of GXYLT1-HA and Shams resulted in an enhancement of the wing vein and margin defects (Figure 3H). Together, these data indicate that Shams and GXYLT1 are functionally homologous, although GXYLT1 seems to be more potent than Shams (see Figure 2H), and suggest that increased xylosylation negatively regulates Notch signaling. Ectopic expression of human XXYLT1-HA resulted in a dramatic loss of margin and thickening of the wing veins, consistent with severe loss of Notch signaling (Figure 3I and Figure S4). In accordance with their enzymatic functions (Figure 1) [22], the XXYLT1-HA overexpression phenotypes are completely suppressed in a *shams^PB/PB^* background with 100% penetrance (Figure 3J; n = 10). This indicates that the observed phenotypes are due to the enzymatic activity of XXYLT1, as they are dependent on Shams to generate xylose-glucose disaccharide substrates. The data also agree with the homology searches which indicate that Shams is the only GXYLT in flies.

To more directly show that the phenotypes caused by overexpressing human xylosyltransferases are due to a loss of Notch signaling, we overexpressed these enzymes along the antero-posterior axis of the developing wing discs by using the *patched (ptc)-GAL4* driver. Again, both enzymes showed phenotypes compatible with loss of Notch signaling in the adult wings, with XXYLT1-HA phenotypes being stronger than the GXYLT1-HA phenotypes (Figure 3K and 3L). Overexpression of XXYLT1-HA resulted in loss of wing margin tissue and a collapse of the L3 and L4 wing veins without affecting proliferation or apoptosis (Figure 3L and S5A–D′). These phenotypes are specific to XXYLT1 overexpression, because they are fully rescued in a *shams* background (Figure S5E). Antibody staining of the third instar wing imaginal discs in these animals showed that the Notch downstream target Cut is either decreased (GXYLT1-HA) or lost (XXYLT1-HA) in the *ptc-GAL4* domain upon human xylosyltransferase overexpression (Figure 3M–3N′). Altogether, these observations indicate that xylosylation negatively regulates Notch signaling.

### EGF Repeats 16–20 of the *Drosophila* Notch Harbor the Functionally Important Sites of Xylosylation

**Figure 4:**
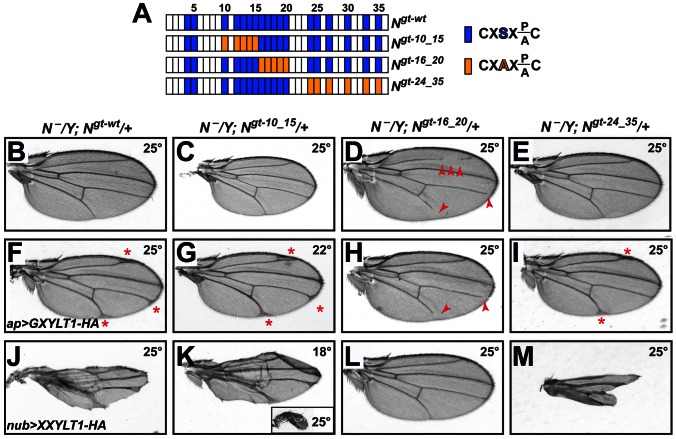
Xylosylation of EGF16–20 negatively regulates *Drosophila* Notch signaling *in vivo*. (A) Schematic of the EGF repeats of wild-type and mutant *Notch* genomic transgenes. Blue boxes show EGF repeats with a consensus *O*-glucosylation site; orange boxes denote EGF repeats with a serine-to-alanine mutation in the *O*-glucosylation site, which prevents the addition of *O*-glucose and therefore xylose. (B–E) *N^−^/Y; N^gt-wt^/+*, *N^−^/Y; N^gt-10_15^/+*, and *N^−^/Y; N^gt-24_35^/+* males exhibit no wing vein loss, but *N^−^/Y; N^gt-16_20^/+* males (D) exhibit loss of L2, L4 and L5 veins (arrowheads). (F) At 25°C, *N^−^/Y; N^gt-wt^/+* males expressing *GXYLT1-HA* in the *apterous-GAL4* domain show thickening of the distal wing veins. (G) In a *N^−^/Y; N^gt-10_15^/+* background, *ap>GXYLT1-HA* becomes lethal at 25°C, and is not suppressed at 22°C. (H) In a *N^−^/Y; N^gt-16_20^/+* background, the *ap>GXYLT1-HA* phenotype is fully suppressed. Note the presence of wing vein loss. (I) *N^gt-24_35^* does not suppress the *ap>GXYLT1-HA* phenotype. (J) At 25°C, *N^−^/Y; N^gt-wt^/+* males expressing *nub>XXYLT1-HA* show severe wing vein and margin defects. (K) The phenotypes are dramatically enhanced in *N^−^/Y; N^gt-10_15^/+* males raised at 25°C (inset) and are comparable to (J) when raised at 18°C. (L,M) The *nub>XXYLT1-HA* phenotypes are fully suppressed in *N^−^/Y; N^gt-16_20^/+* males (L), but are enhanced in *N^−^/Y; N^gt-24_35^/+* males (M; compare to J). All wings, including the inset in M, are shown to scale.

*Notch* transgenes harboring serine-to-alanine mutations in all or most *O*-glucosylation sites show a temperature-sensitive loss of Notch signaling, similar to *rumi* animals [14], [20]. However, when smaller subsets of the *O*-glucosylation sites are mutated and the animals are raised at 25°C or lower, the negative effects of loss of *O*-glucose on Notch is significantly decreased [20]. If loss of xylose on specific EGF repeats results in increased Notch signaling, these mutations should recapitulate the *shams* mutant phenotypes, as loss of *O*-glucose precludes the addition of xylose. To test this, we generated animals that lack endogenous Notch but are rescued by one copy of *Notch* genomic transgenes carrying mutations in various subsets of *O*-glucosylation sites (Figure 4A) [20]. Serine-to-alanine mutations in EGF10–15 or EGF24–35 did not result in loss of wing vein, similar to a wild-type *Notch* transgene (Figure 4B, 4C and 4E). However, *O*-glucose mutations in EGF16–20 resulted in a partial loss of wing veins L2, L4, and L5 (Figure 4D), similar to but somewhat stronger than the *shams* null phenotypes (Figure 2F and 2G). *N^54l9^/Y; N^gt-16_20^/+* animals also exhibited head bristle defects similar to *shams* mutants (Figure S6). These observations nicely match our mass spectrometry data (Figure 1G) and indicate that xylosylation of EGF16–20 plays a negative regulatory role in *Drosophila* Notch signaling.

One prediction from the above conclusion is that mutations in EGF16–20 should suppress the loss of Notch signaling caused by the overexpression of human xylosyltransferases. To test this, we overexpressed GXYLT1-HA and XXYLT1-HA in genetic backgrounds lacking endogenous Notch and rescued by one copy of a wild-type or *O*-glucose mutant *Notch* transgenes. Overexpression of these enzymes in animals with one copy of a wild-type *Notch* transgene raised at 25°C results in phenotypes very similar to their overexpression in a wild-type background raised at the same temperature (Figure 4F and 4J and Figure S4). Among the mutant *Notch* transgenes, *N^gt-16_20^* is the only one which could fully suppress both GXYLT1-HA and XXYLT1-HA overexpression phenotypes (Figure 4G–4I and 4K–4M; n = 10 for each genotype;100% penetrance). Of note, in *N^gt-10_15^* and *N^gt-24_35^* backgrounds the XXYLT1-HA overexpression phenotype is even enhanced, most likely due to the negative effect of loss of these *O*-glucose residues on Notch signaling (Figure 4K and 4M) [20]. Altogether, these data indicate that Shams regulates Notch signaling via an enzymatic mechanism, and that the *O*-glucosylation sites in *Drosophila* Notch EGF16–20 are the biologically-relevant targets of xylosylation by Shams.

### Notch Surface Expression in the Pupal Wing Is Increased upon Loss of Shams or Mutating *O*-glucose Sites in EGF16–20 of Notch

**Figure 5:**
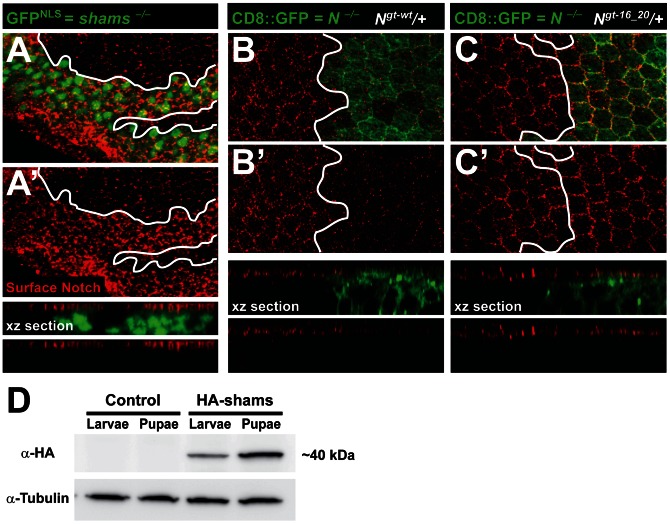
Increased surface expression of Notch in *shams* and *Notchgt-16_20* clones in the pupal wing. All animals were raised at 30°C. (A,A′) Shown are confocal images from a pupal wing at 22–24 hours after puparium formation (APF) with a MARCM clone of *shams^Δ34^* marked by nuclear GFP (GFP^NLS^). Surface expression of Notch is shown in red. Note, also in the xz section, that the Notch surface level at this stage is increased in *shams* mutant cells. (B–C′) Shown are confocal images of pupal wings around 22 hours APF from animals harboring MARCM clones of the *Notch^54l9^* protein-null allele (marked by CD8::GFP) with one copy of either a wild-type *Notch* transgene (B,B′) or a *Notch* transgene with *O*-glucose mutations in EGF16–20 (C,C′). The only source of Notch in the clones is the *Notch* transgene. Note, also in xz sections, that the level of surface Notch in clones harboring the *Notch^gt-16_20^* is increased compared to that in clones harboring *Notch^gt-wt^*. (D) Anti-HA Western blot on larval and pupal protein extracts from animals harboring one copy of an HA-tagged Shams genomic transgene (HA-Shams; *shams^gt-wt-HA^-attVK22*) or *attVK22* control animals. Tubulin was used as loading control. Pupal extracts show relatively higher levels of HA-Shams.

To examine the effects of loss of *shams* on Notch localization, we performed Notch surface staining on larval wing imaginal discs and pupal wings harboring *shams* mutant clones. Loss of *shams* does not affect the surface expression of Notch in third instar wing imaginal disc (Figure S7A–A″). However, more Notch protein is present in and at the surface of *shams* mutant cells in the pupal wing (Figure 5A and 5A′ and Figure S7B–B′). We also sought to determine whether mutating the *O*-glucose sites on EGF16–20 of Notch results in increased cell surface levels of Notch. To this end, we generated Mosaic Analysis with a Repressible Cell Marker (MARCM) clones [28] of a protein-null allele of *Notch* in a background harboring one copy of the wild-type or an EGF16–20 mutant *Notch*, similar to what we have described before for other mutant *Notch* transgenes [20]. In these animals, heterozygous cells have both endogenous *Notch* and a copy of our transgene, but cells in the mutant clones only harbor a copy of the transgene. Accordingly, the level of Notch expressed from the wild-type transgene in the clones is less than that in the heterozygous cells (Figure 5B and 5B′), in agreement with our previous report [20]. Clones of *Notch^gt-16_20^* in larval wing disc do not show an increase in Notch surface expression (data not shown). However, in the pupal wing, the level of surface Notch expressed from the *Notch^gt-16_20^* transgene in the clones is significantly increased compared to that expressed from the wild-type *Notch^gt-wt^* transgene (Figure 5C and 5C′; compare to 5B′). One potential explanation for the difference between the effects of loss of Notch xylosylation in pupae versus larvae could be different levels of Shams expression at these stages. Indeed, Western blot confirmed higher Shams expression in pupae compared to third instar larvae (Figure 5D). In agreement with a role for xylose in surface expression of Notch, overexpression of human XXYLT1 results in a significant decrease and overexpression of human GXYLT1 results in a mild and partially penetrant decrease in Notch surface expression in the larval wing imaginal discs (Figure S8). Altogether, these observations suggest that addition of xylose residues to EGF16–20 of Notch decreases the availability of Notch at the cell surface.

## Discussion

Our data indicate that there are significant differences between the ways *O*-glucose monosaccharides and their extended (xylosylated) form regulate *Drosophila* Notch signaling. First, *O*-glucosylation promotes Notch signaling [14], [20], but xylosylation inhibits Notch signaling. Secondly, loss of *O*-glucosylation affects Notch signaling in all contexts studied so far [14], [20], [29], but loss of xylosylation only affects Notch signaling in certain contexts, i.e. wing vein development and head bristle formation. Finally, *O*-glucose residues on all EGF repeats contribute to the Notch signal strength in redundant and/or additive fashions [20], but xylose residues seem to be only required on a subset of Notch EGF repeats. In other words, unlike glucose residues which function globally on the Notch extracellular domain to promote Notch signaling in various contexts [14], [20], [29], xylose residues function locally on a specific region of Notch to decrease signaling in certain contexts. Thus, our data show that the strength of the Notch signaling pathway can be fine-tuned by altering the distribution and relative levels of *O*-glucose monosaccharide and their xylose-containing extended forms on the Notch receptor. Since xylose-xylose-glucose glycans on EGF repeats are the only known glycans in animals with a terminal xylose [30], [31], our data strongly suggest that at least in *Drosophila*, terminal xylose residues play a fairly specific role in regulating the Notch signaling pathway.

Our observations provide compelling evidence that the negative effects of xylosyltransferases on *Drosophila* Notch signaling are primarily mediated via their enzymatic activity. First, serine-to-alanine mutations in the *O*-glucosylation sites in EGF16–20 of the *Drosophila* Notch recapitulate the wing vein and head bristle phenotypes of *shams*. Since loss of the protein *O*-glucosyltransferase Rumi results in loss of Notch signaling [14], [20], the observed gain of Notch signaling phenotypes cannot be due to loss of *O*-glucose from these EGF repeats, but are very likely due to the loss of xylose normally attached to the *O*-glucose. Secondly, mutating the *O*-glucose sites of EGF16–20 fully suppresses the Notch loss-of-function phenotypes caused by GXYLT1 and XXYLT1 overexpression, strongly suggesting that these phenotypes result from the addition of xylose by these enzymes to *O*-glucose on these EGF repeats. Lastly, although the Notch loss-of-function phenotypes of XXYLT1 overexpression are much more severe than those caused by GXYLT1 overexpression, loss of *shams* fully suppresses the XXYLT1 overexpression phenotypes but only partially suppresses the GXYLT1 overexpression phenotype. These data fully match the enzymatic activities of these proteins: GXYLT1 and Shams both add the first xylose to *O*-glucose and function in parallel, but XXYLT1 adds the second xylose and therefore completely depends on the activity of GXYLT1/Shams.

A hallmark of Notch receptors is the presence of many EGF repeats in their extracellular domain. However, the functional importance of only a handful of Notch EGF repeats has been elucidated. Specifically, EGF11 and 12 are necessary for ligand-binding [32], EGF 24, 25, 27 and 29 negatively regulate Notch signaling, likely through opposing ligand binding [33], and EGF8 is required for binding of Notch to Serrate but not Delta [34]. By showing that EGF16–20 (or a subset of them) negatively regulate *Drosophila* Notch signaling in a xylose-dependent manner, our data assign a function to these EGF repeats. Loss of *shams* or loss of xylosylation on EGF16–20 affects surface expression of Notch in the pupal wing and results in defects in wing vein formation, a process primarily regulated in the pupal stage. In contrast, loss of *shams* does not affect Notch expression in the larval wing discs, or the wing margin formation, which primarily occurs at the larval stage. Together, these observations suggest that Notch signaling in the pupal wing is more sensitive to loss of xylosylation compared to the larval wing disc, likely because of the relatively higher levels of Shams expression in the pupal stage. Nevertheless, given the loss of Notch signaling observed upon GXYLT1 and XXYLT1 overexpression, the larval wing discs likely have the machinery required to recognize and respond to xylose on Notch. Increased availability of Notch at the cell surface of *shams* and *Notch^gt-16_20^* mutant clones in the pupal wing suggests a molecular mechanism for the role of xylose in regulating Notch signaling. However, it remains to be seen whether other steps of Notch signaling, including ligand-binding and *cis*-inhibition are also affected by the loss of xylose.

Although cell type variability exists, all *O*-glucosylated EGF repeats of the mouse Notch1 harbor xylose-xylose-glucose trisaccharides at high stoichiometry [19]. Our previous work [14] and current mass spectrometry experiments indicate that all of the EGF repeats of the *Drosophila* Notch with a consensus Rumi target motif analyzed thus far (16 out of 18) harbor *O*-glucose. However, we find that xylose is only added to a subset of *O*-glucosylated Notch EGF repeats in *Drosophila*. This difference might result from different efficiency of the mammalian versus *Drosophila* xylosyltransferases *in vivo* despite their similar *in vitro* levels of activity (Figure 1E). Indeed, overexpression of human GXYLT1, but not its *Drosophila* homolog Shams, results in Notch loss-of-function phenotypes in the wing, suggesting that GXYLT1 is more potent in adding xylose to Notch *in vivo*. Moreover, the difference between the distribution of (xylose)-xylose-glucose saccharides on fly and mammalian Notch could in part be due to the presence of two GXYLT enzymes in mammals instead of only one (Shams) in flies. Regardless of the mechanisms underlying the observed differences, it will be of great interest to determine whether alteration of the level or distribution of xylose-xylose-glucose saccharides on Notch receptors can modulate the strength of mammalian Notch signaling as well.

## Materials and Methods

### *Drosophila* Strains and Genetics

The following strains were used in this study: *y w*, *y w; D/TM6, Tb^1^*, *w; noc^Sco^/CyO*, *w; noc^Sco^/CyO; TM3, Sb/TM6, Tb^1^*, *w; CyO, P{FRT(w^+^)Tub-PBac\T}2/wg^Sp-1^*, *y w hsFLP; Dr/TM3*, *apterous-GAL4*, *patched-GAL4*, *y w N^54l9^ FRT19A/FM7*, *N^55e11^/FM7c*, *N^Ax-E2^*, *Df(3R)BSC494/TM6C, Sb^1^*, *w; PBac{RB}CG9996^01256^*, *attVK22*, *UAS-CG8::GFP* (Bloomington Stock Center), *w; PBac{RB}CG11836^e01985^* (Exelixis), *shams^rev^*, *shams^Δ34^/TM6, Tb^1^*, *shams^gt-wt^-attVK22*, *shams^gt-wt-HA^-attVK22*, *CG11836^gt-wt^-attVK22, UASattB-shams-HA-VK22*, *UASattB-GXYLT1-HA-VK22*, *UASattB-XXYLT1-HA-VK22, Ubx-FLP FRT19A/FM7; Act-GAL4 UAS-CD8::GFP/CyO* (this study), *N^gt-wt^-attVK22*, *N^gt-10_15^-attVK22*, *N^gt-16_20^-attVK22*, *N^gt-24_35^-attVK22* [20], *y w Ubx-FLP tub-GAL4 UAS-GFP^nls^-6X-Myc; FRT82B y^+^ tub-GAL80/TM6, Ubx* [14], *vas-int-ZH-2A; attVK22* [35], *nubbin-GAL4* (Georg Halder). All crosses were performed on standard media and incubated at described temperatures.

To test the effect of *Notch* mutations on the adult wing, *w; N^gt-wt^*, *N^gt-10_15^*, *N^gt-16_20^*, or *N^gt-24_35^* males were crossed to *N^54l9^/FM7*, *B* females. *B^+^* male progeny were scored for wing and head bristle defects. To determine the effect of *Notch* mutations on GXYLT1-HA and XXYLT1-HA overexpression in the wing, *B^+^*, *Cy^+^* male progeny were scored from *apterous-GAL4 UAS-GXYLT1-HA/CyO* or *nubbin-GAL4 UAS-XXYLT1-HA/CyO* males crossed with *N^54l9^/FM7; N^gt-wt^/+*, *N^54l9^/FM7; N^gt-10_15^/+*, *N^54l9^/FM7; N^gt-16_20^/+*, or *N^54l9^/FM7; N^gt-24_35^/+* females. To generate MARCM clones of *Notch* harboring a *Notch* genomic transgene, *Ubx-FLP FRT19A/Y; Act-GAL4 UAS-CD8::GFP/CyO* males were crossed to *Notch^54l9^/FM7; N^gt-wt^/+* or *N^54l9^/FM7; N^gt-16_20^/+* females. All MARCM crosses were set at room temperature and transferred to 30°C at L1–L2 instar stage. For further details on *Drosophila* genetics and other techniques used in this study, please see Text S1.

### Glycosyltransferase Assays

For recombinant expression of Shams (CG9996) the predicted C-terminal lumenal domain starting from Gln23 was amplified from *Drosophila w^1118^* cDNA and cloned into the *pFast-Bac1* vector (Invitrogen) encoding the HBM-secretion signal followed by the Protein A coding sequence, as described for human GXYLT1 [23]. Constructs were expressed in Sf9 insect cells using the Bac-to-Bac System (Invitrogen) and secreted proteins were purified by IgG-Sepahrose-6 Fast Flow beads (GE-Healthcare) as described [23]. Activity assays were performed on bead-coupled enzyme in the presence of radiolabeled UDP-sugars as described before [23], except that samples were incubated for 2 h at 27°C. To measure activity on EGF repeats, the Notch EGF16–20 fragment was expressed in Sf9 insect cells, purified by Nickel affinity chromatography, and used in an *in vitro* assay followed by mass spectrometric analysis as described [22].

### *O*-Glucose Site Mapping on *Drosophila* Notch EGF1–36 Expressed in S2 Cells

A construct encoding EGF1–36 from *Drosophila* Notch with a C-terminal 3X-FLAG tag (EGF1–36-FLAG_3_, generously provided by Dr. Ken Irvine) was expressed in *Drosophila* S2 cells. EGF1–36-FLAG_3_ was purified from medium, reduced and alkylated, and subjected to in-gel protease digests as described [36]. *O*-Glucose modified glycopeptides were identified by neutral loss of the glycans during collision-induced dissociation (CID) using nano-LC-MS/MS as described [19].

### Dissections, Staining, Image Acquisition and Processing

Dissection and staining were performed by using standard methods. For surface staining, third instar larval imaginal discs and pupal wings were dissected and incubated with anti-Notch antibody in the absence of detergent. Antibodies used were mouse α-Cut (2B10) 1∶500, mouse anti-Notch (C458.2H) 1∶100, mouse α-Delta (C594.9B) 1∶100 (Developmental Studies Hybridoma Band), goat α-HA 1∶50 (GenScript), mouse α-phosphorylated Histone H3 (Ser10) 1∶100, rabbit α-Cleaved Caspase 3 (Asp175) 1∶50 (Cell Signaling), donkey-α-goat-Dylight549 1∶500, donkey-α-mouse-Cy5 1∶500, goat-α-mouse-Cy3 1∶500, donkey-α-Rabbit-Cy5 1∶500, donkey-α-Guinea Pig-Cy3 1∶500 (Jackson ImmunoResearch Laboratories). Confocal images were scanned using a Leica TCS-SP5 microscope and processed with Amira5.2.2. Images were processed with Adobe Photoshop CS5; Figures were assembled in Adobe Illustrator CS5.

### Western Blots

Third instar larvae and pupae aged 22 hours after puparium formation (APF) were collected from *VK22* control animals or animals harboring one copy of the *shams^gt-wt-HA^* genomic transgene. Protein extracts were generated using RIPA buffer (Boston BioProducts) and a Protease Inhibitor Cocktail (Roche). Protein extracts were separated on 12% acrylamide gel and transferred to PVDF membrane. Blots were probed with goat α-HA 1∶300 (GenScript), mouse α-Tubulin 1∶1000 (Santa Cruz Biotech), donkey-α-goat-HRP 1∶2000 and goat α-mouse-HRP 1∶2000 antibodies (Jackson ImmunoResearch Laboratories). Western blots were developed using Pierce ECL Western Blotting Substrates (Thermo Scientific) and imaged with an LAS4000 GE ImageQuant Imager. Three independent experiments showed the same result.

## Supporting Information

Figure S1 **CG9996 (Shams) is the only close homolog of human GXYLT1/2 in** ***Drosophila*** **.** (A) Protein domain structure of *Drosophila* and human xylosyltransferases and their *Drosophila* homologs CG9996 (Shams) and CG11388. TM, transmembrane domain. (B) Percent amino acid identity among GXYLT1/2 and XXYLT1 and their *Drosophila* homologs. Upper right cells indicate overall identities and lower left cells indicate sequence identity in the putative catalytic domain. Note that GXYLT1, GXYLT2 and CG9996 (Shams) fall into one group (green), whereas XXYLT1 and CG11388 fall into the other (orange) based on the level of sequence identity. (C) Protein sequence comparison of CG9996 (Shams) and human GXYLT1/2. Conserved amino acids are highlighted in black, and the metal binding DxD-like motifs, which are found in a large number of glycosyltransferases, are highlighted in red.
(TIF)

Figure S2 **Although predicted** ***O*** **-glucose sites are modified with** ***O*** **-glucose, only a subset is elongated by xylose.** *Drosophila* Notch EGF1–36-FLAG_3_ was expressed in *Drosophila* S2 cells, purified from the medium, digested with proteases, and analyzed by nano-LC-MS/MS to identify *O*-glucosylated peptides [19]. (A–F) For each peptide, an MS spectrum, showing the selection of the parent ion for fragmentation (top), and an MS/MS spectrum, showing the resulting CID fragmentation (bottom), are presented. Ions in the MS/MS spectrum showing losses of the modifications are indicated, and the EGF repeat from which the peptide is derived is labeled above each MS spectrum. Note that some sites are only modified with *O-*glucose monosaccharide (*e.g.* EGF10 (A) and EGF35 (F)), some with *O*-glucose disaccharide (*e.g.* EGF14 (B), EGF15 (C) and EGF19 (E)), and only two have been found with *O*-glucose trisaccharide (EGF16 (Figure 1F) and EGF18 (D)). While several EGF repeats are modified with more than one form of *O*-glucose, only spectra showing the most elongated *O*-glucose saccharide detected at any individual site are shown. Representative spectra are shown here and in Figure 1F. Additional spectra will be presented in a separate publication (Rana *et al*., in preparation). Figure 1G shows a summary of the *O*-glucose site mapping data.
(TIF)

Figure S3 **Loss of** ***shams*** **suppresses the** ***N^55e11^*** **haploinsufficient phenotype.** The percentage of *N^55e11/+^* wings exhibiting vein defects at 25° and 30°C is decreased in a temperature-sensitive manner in the absence of *shams*. Note that at 30°C, the wing vein phenotype is rescued in ∼98% of the wings (n = 34), whereas at 25°C the wing vein phenotype is rescued in ∼48% of the wings (n = 46).
(TIF)

Figure S4 **Overexpression of human xylosyltransferases inhibits Notch signaling.** (A) Wing-specific overexpression of HA-tagged human GXYLT1 by *apterous-GAL4* (*ap>GXYLT1-HA*) induces thickening of the distal ends of wing veins at 25°C (asterisks). (B) Overexpression of XXYLT1-HA by *nubbin-GAL4* (*nub>XXYLT1-HA*) results in severe wing vein and margin defects at 25°C.
(TIF)

Figure S5 **Overexpression of human XXYLT1 does not affect cell proliferation and cell death.** All animals were raised at 30°C. (A,A′) A control disc expressing CD8::GFP by *patched-GAL4 (ptc>GFP)* shows scattered labeling of phosphorylated histone H3 (PH3). (B–B′) Overexpression of XXYLT1-HA by *patched-GAL4* (*ptc>XXYLT1-HA*) does not alter the distribution of the PH3-positive cells. (C,C′) Minimal levels of activated Caspase 3 (Casp3^*^) are present in *ptc>GFP* control wing discs. (D,D′) No change in activated Caspase 3 levels are observed upon *ptc>XXYLT1-HA* overexpression. (E) The wing margin and vein defects of *ptc>XXYLT1-HA* flies are suppressed in a *shams* ^Δ*34/PB*^ background (compare to Figure 3L).
(TIF)

Figure S6 **Expression of** ***N^gt-16_20^*** **results in the loss of head bristles, similar to** ***shams*** **mutants.** *N^−^/Y; N^gt-16_20^/+* males raised at 30°C exhibit loss of head bristles.
(TIF)

Figure S7 **Notch and Delta expression in** ***shams*** **clones.** MARCM clones of *shams^Δ34^* are marked by nuclear GFP (GFP^NLS^). All animals were raised at 30°C. (A–A″) Loss of *shams* does not affect the surface expression of Notch in third instar wing imaginal discs. Surface expression of Notch is shown in red. Note, also in the xz section, that the Notch surface level at this stage is not affected by the loss of *shams*. (B–B″) Loss of *shams* in a pupal wing at 22 hours after puparium formation (APF) results in an increase in total Notch expression but does not alter Delta expression.
(TIF)

Figure S8 **Surface expression of Notch is decreased upon xylosyltransferase overexpression.** All animals were raised at 30°C. Cells overexpressing the HA-tagged xylosyltransferases are to the left of the white line in each panel. (A) Domain specific expression of an HA-tagged GXYLT1 using *patched-GAL4 (ptc>GXYLT1-HA)* resulted in a mild decrease in surface expression of Notch. This decrease is Notch surface expression is not completely penetrant. (B) Overexpression of XXYLT1-HA using *patched-GAL4* (*ptc>XXYLT1-HA*) resulted in a severe decrease of Notch at the cell surface.
(TIF)

Text S1 **Contains additional details on** ***Drosophila*** **genetics, obtaining adult** ***Drosophila*** **images, molecular biology, and glycosyltransferase assays.**
(DOCX)
