# Supplementary figures and images for: Negative Regulation of Notch Signaling by Xylose
Source: PLoS Genet. 2013 Jun 6;9(6):e1003547. doi: 10.1371/journal.pgen.1003547 (PMC3675014; doi:10.1371/journal.pgen.1003547)

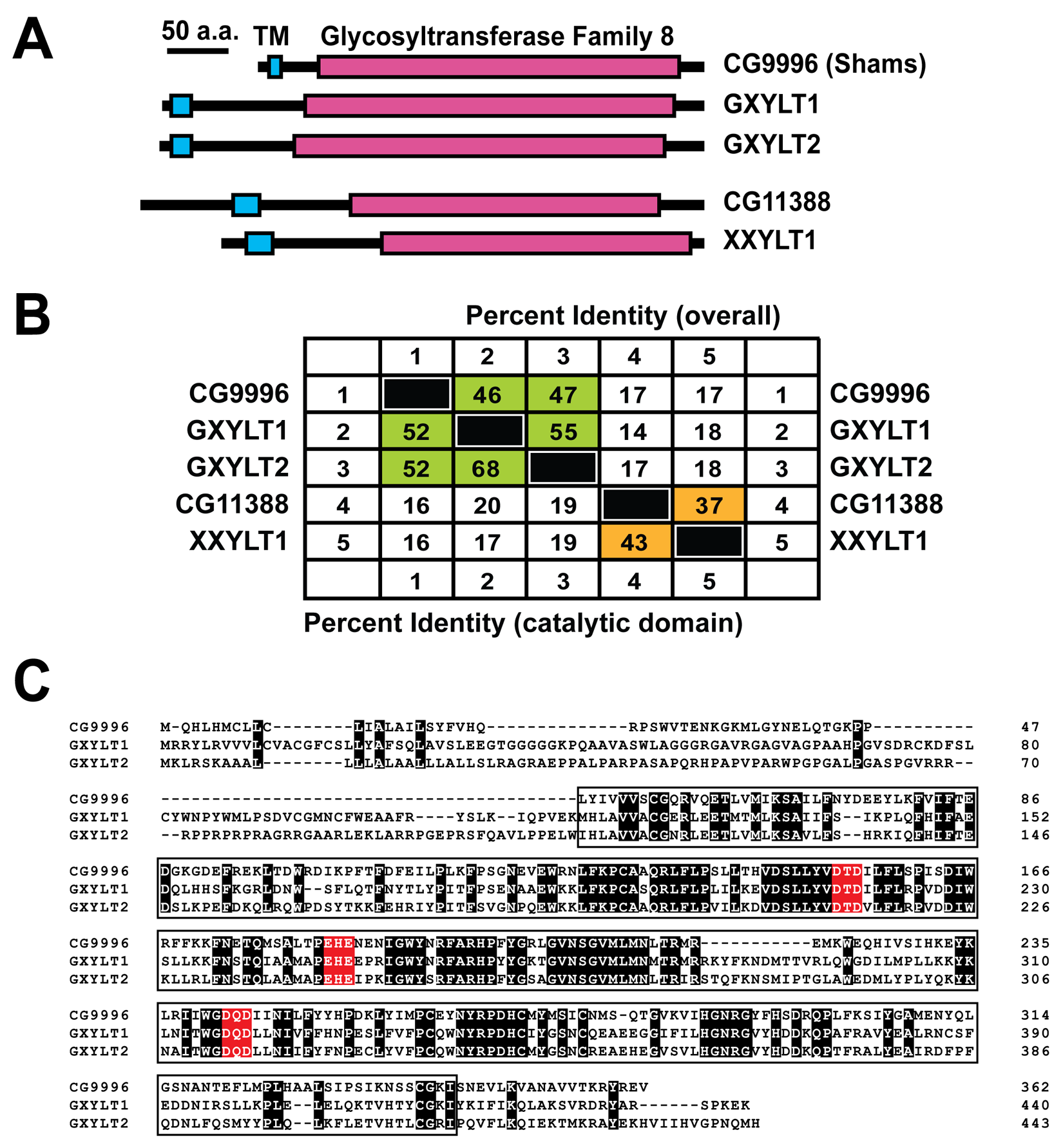

Supplement: Figure S1 — CG9996 (Shams) is the only close homolog of human GXYLT1/2 in Drosophila . (A) Protein domain structure of Drosophila and human xylosyltransferases and their Drosophila homologs CG9996 (Shams) and CG11388. TM, transmembrane domain. (B) Percent amino acid identity among GXYLT1/2 and XXYLT1 and their Drosophila homologs. Upper right cells indicate overall identities and lower left cells indicate sequence identity in the putative catalytic domain. Note that GXYLT1, GXYLT2 and CG9996 (Shams) fall into one group (green), whereas XXYLT1 and CG11388 fall into the other (orange) based on the level of sequence identity. (C) Protein sequence comparison of CG9996 (Shams) and human GXYLT1/2. Conserved amino acids are highlighted in black, and the metal binding DxD-like motifs, which are found in a large number of glycosyltransferases, are highlighted in red. (TIF) [file pgen.1003547.s001.tif]

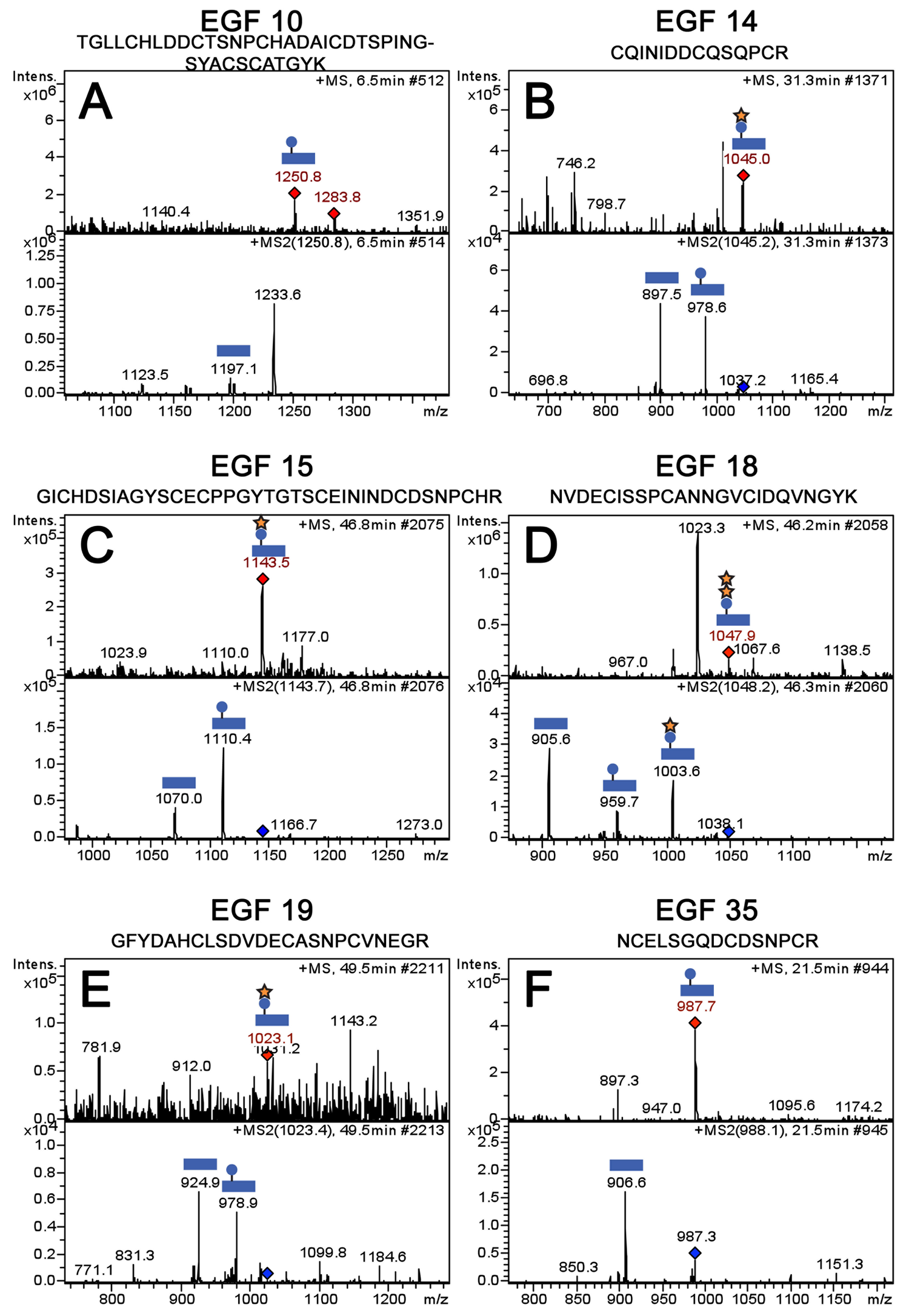

Supplement: Figure S2 — Although predicted O -glucose sites are modified with O -glucose, only a subset is elongated by xylose. Drosophila Notch EGF1–36-FLAG3 was expressed in Drosophila S2 cells, purified from the medium, digested with proteases, and analyzed by nano-LC-MS/MS to identify O-glucosylated peptides [19]. (A–F) For each peptide, an MS spectrum, showing the selection of the parent ion for fragmentation (top), and an MS/MS spectrum, showing the resulting CID fragmentation (bottom), are presented. Ions in the MS/MS spectrum showing losses of the modifications are indicated, and the EGF repeat from which the peptide is derived is labeled above each MS spectrum. Note that some sites are only modified with O-glucose monosaccharide (e.g. EGF10 (A) and EGF35 (F)), some with O-glucose disaccharide (e.g. EGF14 (B), EGF15 (C) and EGF19 (E)), and only two have been found with O-glucose trisaccharide (EGF16 (Figure 1F) and EGF18 (D)). While several EGF repeats are modified with more than one form of O-glucose, only spectra showing the most elongated O-glucose saccharide detected at any individual site are shown. Representative spectra are shown here and in Figure 1F. Additional spectra will be presented in a separate publication (Rana et al., in preparation). Figure 1G shows a summary of the O-glucose site mapping data. (TIF) [file pgen.1003547.s002.tif]

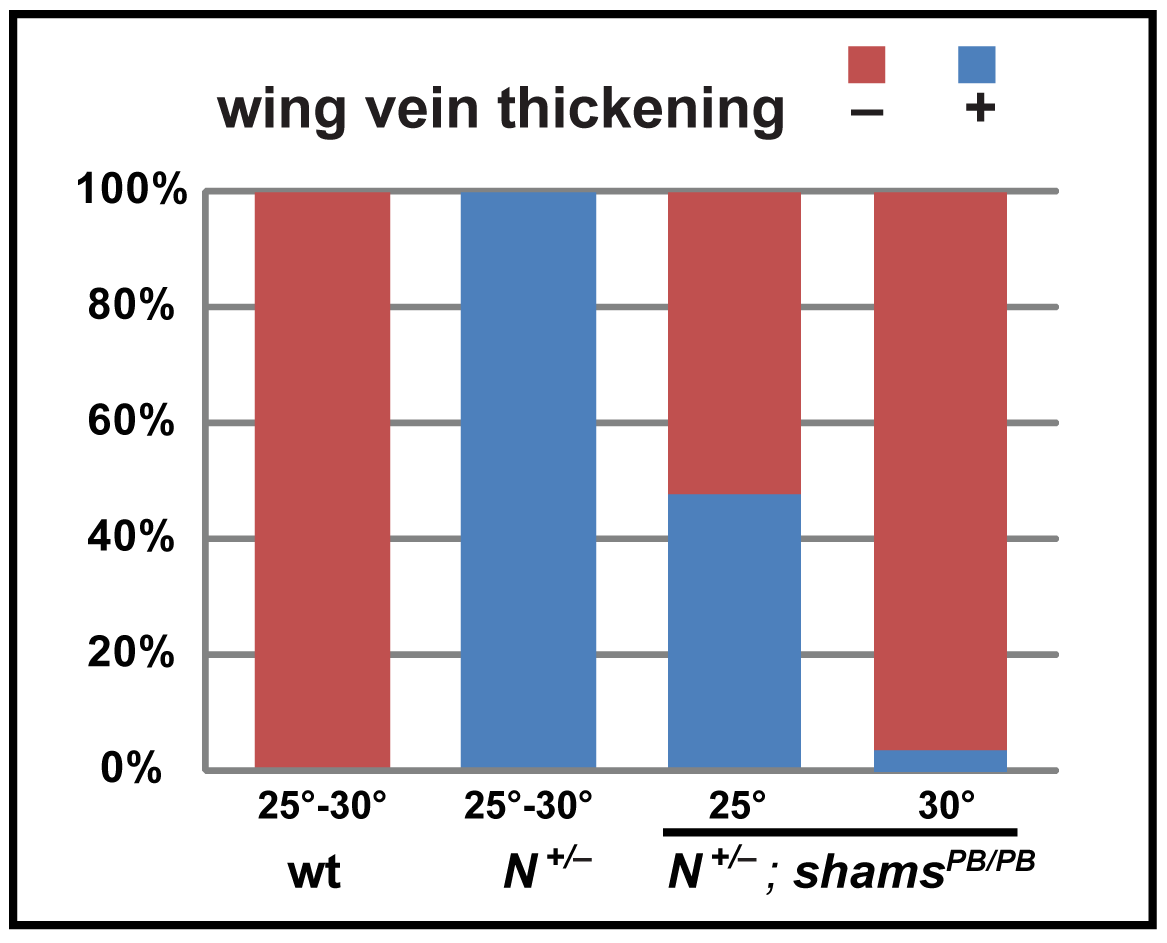

Supplement: Figure S3 — Loss of shams suppresses the N55e11 haploinsufficient phenotype. The percentage of N55e11/+ wings exhibiting vein defects at 25° and 30°C is decreased in a temperature-sensitive manner in the absence of shams. Note that at 30°C, the wing vein phenotype is rescued in ∼98% of the wings (n = 34), whereas at 25°C the wing vein phenotype is rescued in ∼48% of the wings (n = 46). (TIF) [file pgen.1003547.s003.tif]

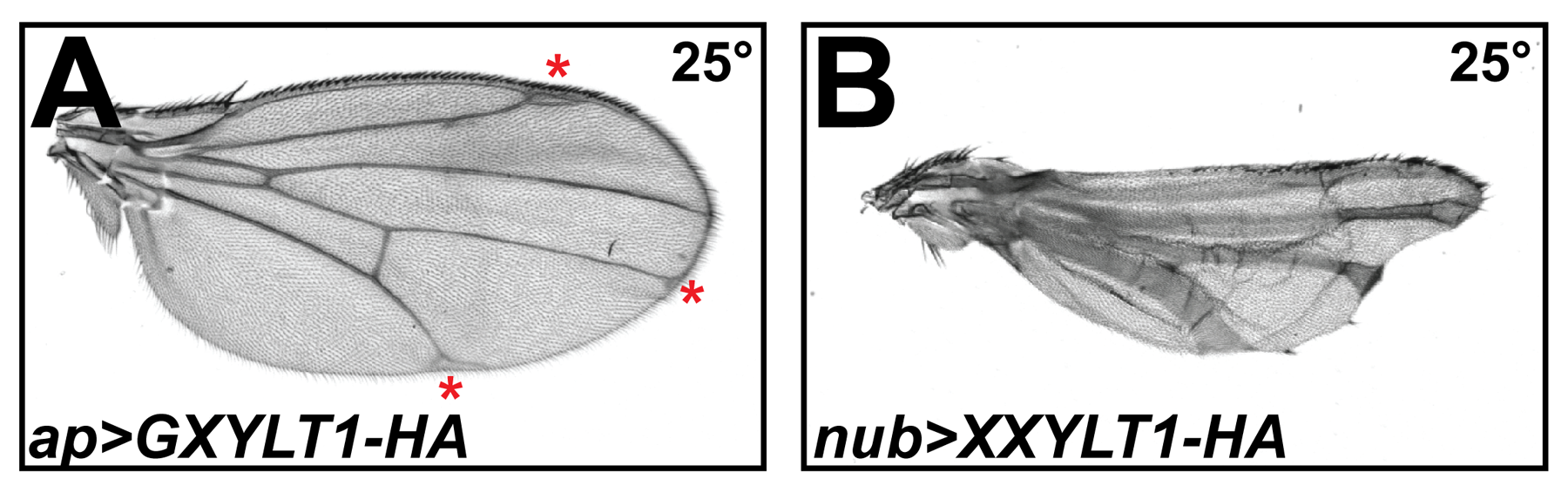

Supplement: Figure S4 — Overexpression of human xylosyltransferases inhibits Notch signaling. (A) Wing-specific overexpression of HA-tagged human GXYLT1 by apterous-GAL4 (ap>GXYLT1-HA) induces thickening of the distal ends of wing veins at 25°C (asterisks). (B) Overexpression of XXYLT1-HA by nubbin-GAL4 (nub>XXYLT1-HA) results in severe wing vein and margin defects at 25°C. (TIF) [file pgen.1003547.s004.tif]

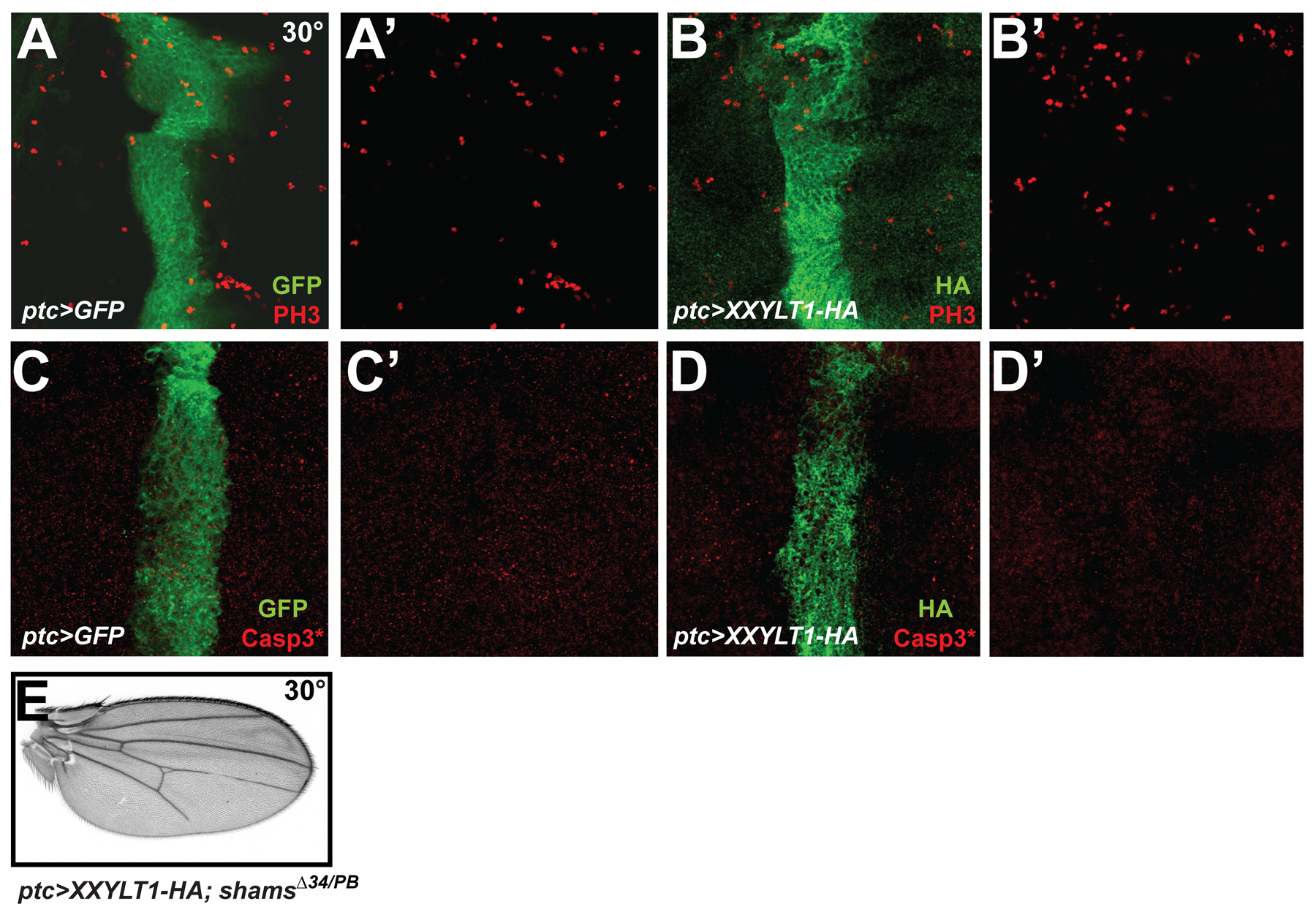

Supplement: Figure S5 — Overexpression of human XXYLT1 does not affect cell proliferation and cell death. All animals were raised at 30°C. (A,A′) A control disc expressing CD8::GFP by patched-GAL4 (ptc>GFP) shows scattered labeling of phosphorylated histone H3 (PH3). (B–B′) Overexpression of XXYLT1-HA by patched-GAL4 (ptc>XXYLT1-HA) does not alter the distribution of the PH3-positive cells. (C,C′) Minimal levels of activated Caspase 3 (Casp3*) are present in ptc>GFP control wing discs. (D,D′) No change in activated Caspase 3 levels are observed upon ptc>XXYLT1-HA overexpression. (E) The wing margin and vein defects of ptc>XXYLT1-HA flies are suppressed in a shams Δ34/PB background (compare to Figure 3L). (TIF) [file pgen.1003547.s005.tif]

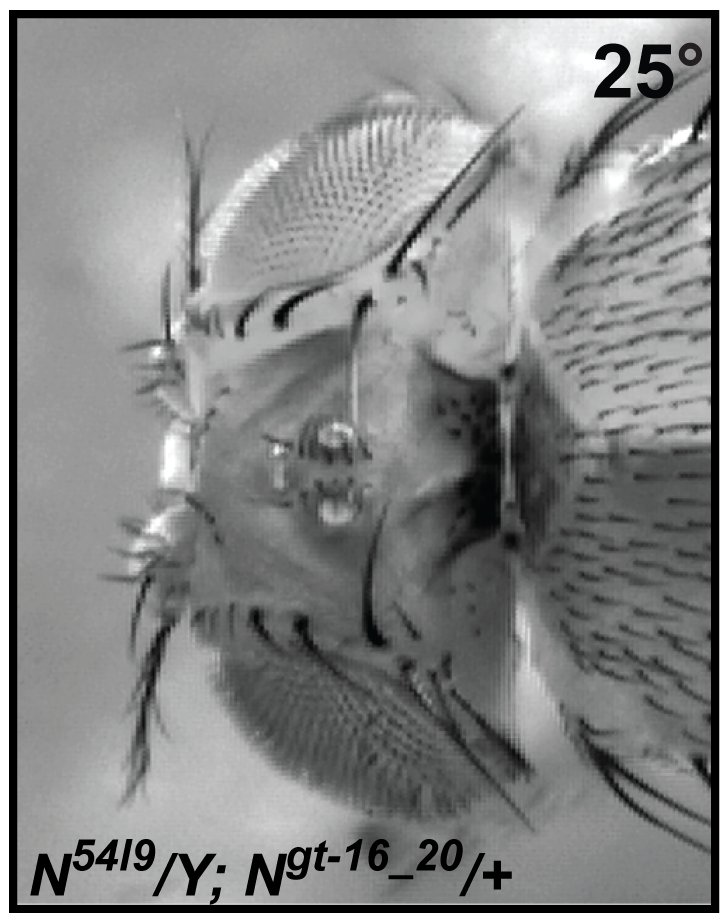

Supplement: Figure S6 — Expression of Ngt-16_20 results in the loss of head bristles, similar to shams mutants. N−/Y; Ngt-16_20/+ males raised at 30°C exhibit loss of head bristles. (TIF) [file pgen.1003547.s006.tif]

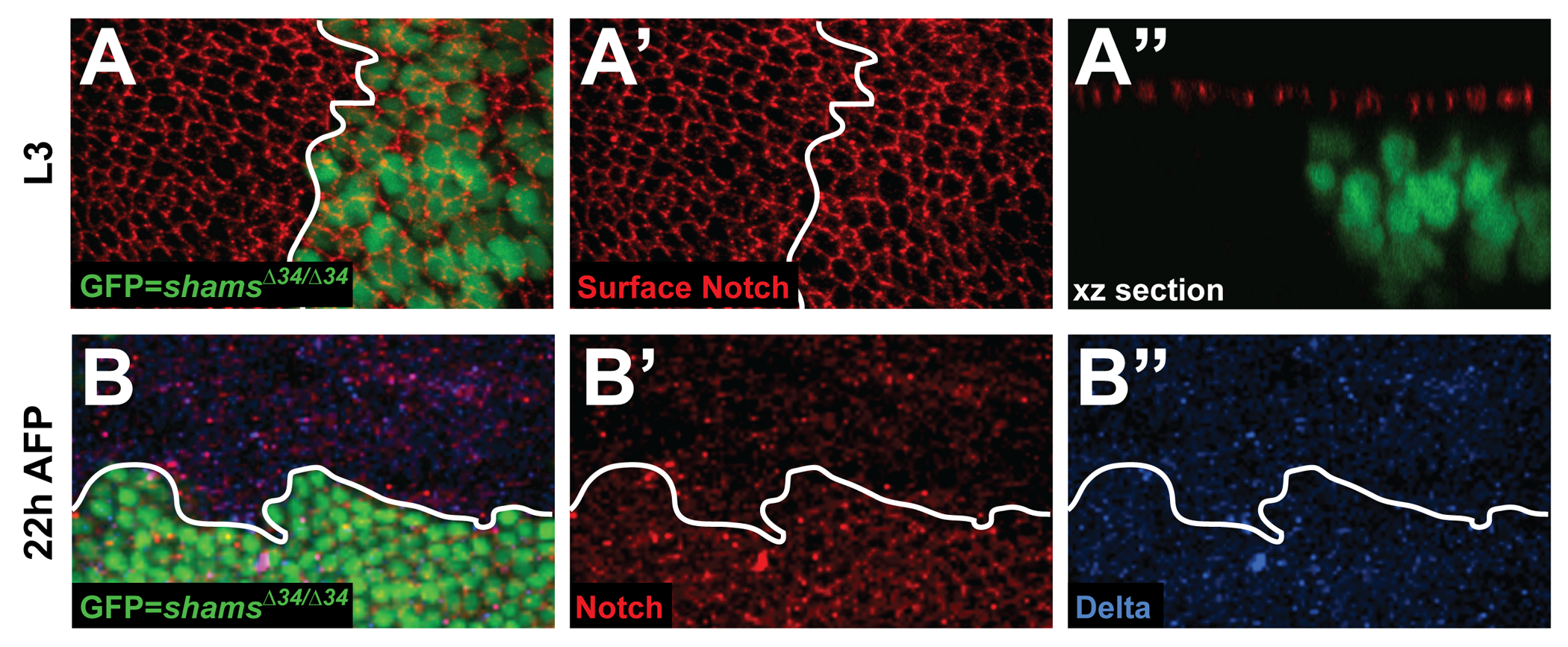

Supplement: Figure S7 — Notch and Delta expression in shams clones. MARCM clones of shamsΔ34 are marked by nuclear GFP (GFPNLS). All animals were raised at 30°C. (A–A″) Loss of shams does not affect the surface expression of Notch in third instar wing imaginal discs. Surface expression of Notch is shown in red. Note, also in the xz section, that the Notch surface level at this stage is not affected by the loss of shams. (B–B″) Loss of shams in a pupal wing at 22 hours after puparium formation (APF) results in an increase in total Notch expression but does not alter Delta expression. (TIF) [file pgen.1003547.s007.tif]

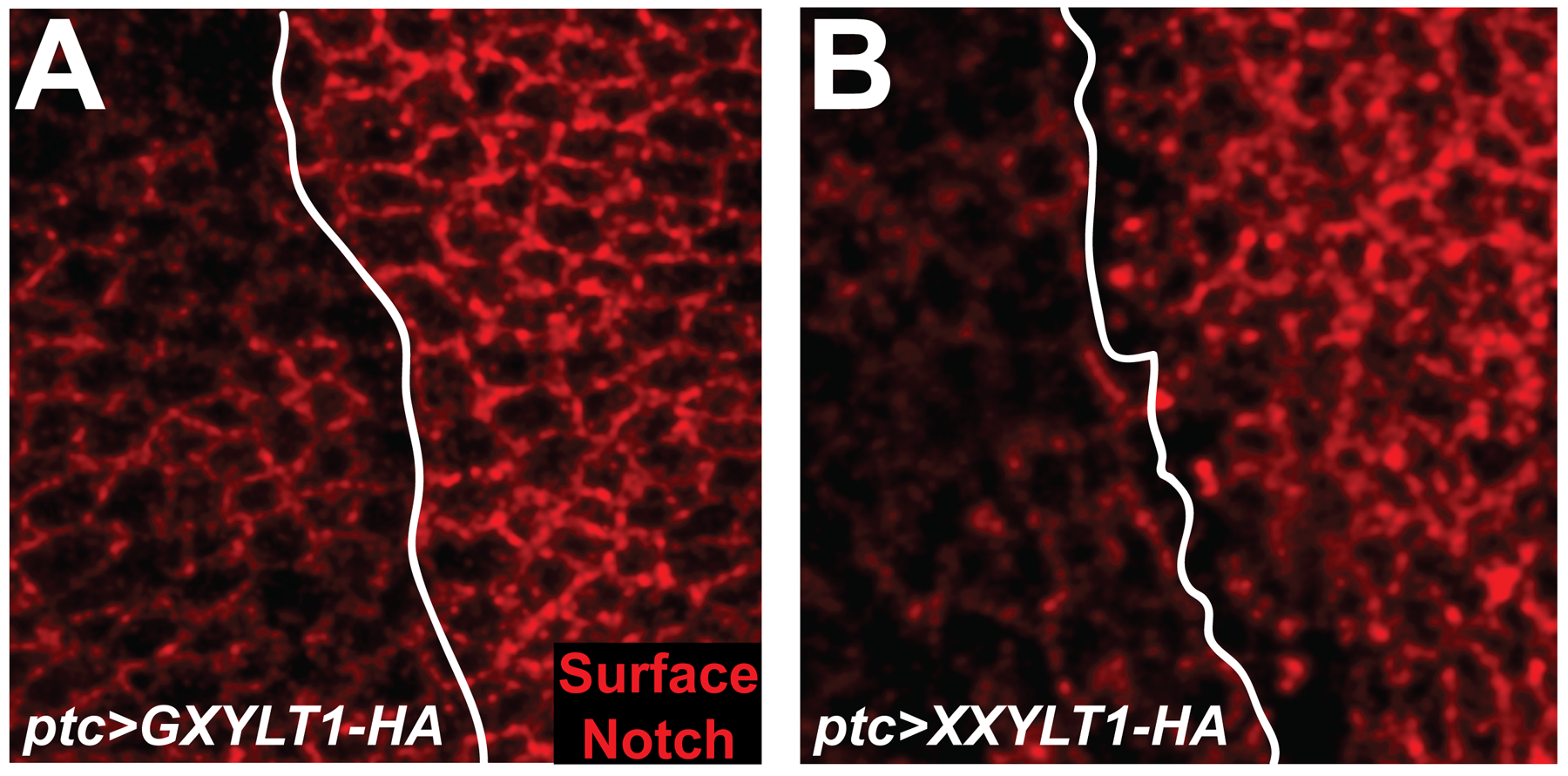

Supplement: Figure S8 — Surface expression of Notch is decreased upon xylosyltransferase overexpression. All animals were raised at 30°C. Cells overexpressing the HA-tagged xylosyltransferases are to the left of the white line in each panel. (A) Domain specific expression of an HA-tagged GXYLT1 using patched-GAL4 (ptc>GXYLT1-HA) resulted in a mild decrease in surface expression of Notch. This decrease is Notch surface expression is not completely penetrant. (B) Overexpression of XXYLT1-HA using patched-GAL4 (ptc>XXYLT1-HA) resulted in a severe decrease of Notch at the cell surface. (TIF) [file pgen.1003547.s008.tif]
