# Supplementary material for: Negative Regulation of Notch Signaling by Xylose
Source: PLoS Genet. 2013 Jun 6;9(6):e1003547. doi: 10.1371/journal.pgen.1003547 (PMC3675014; doi:10.1371/journal.pgen.1003547)
Supplement: Text S1 — Contains additional details on Drosophila genetics, obtaining adult Drosophila images, molecular biology, and glycosyltransferase assays. (DOCX) [file pgen.1003547.s009.docx]

**Text S1**

**Supporting Materials and Methods**

***Drosophila* Genetics.** Fly strains harboring the *VK22* docking site were used for injections to generate the genomic and overexpression transgenic animals, and transformation was performed using φC31-mediated transgenesis [[1](#_ENREF_1)].

To excise the *piggyBac* insertion and revert *shams^e01256/e01256^*, *w; CyO, {FRT(w^+^)Tub-PBac\T}2/+; PBac{RB}CG9996^01256^*/*+* males were crossed to *y w; D/TM6, Tb* females. White-eyed male progeny were used to generate candidate revertant fly lines. Genomic PCR with primers that flank the excision and complementation tests with *PBac{RB}CG9996^01256^* animals at 25°and 30°C were used to confirm the revertant allele.

To generate the *shams^Δ34^* null allele, FLP/FRT-mediated recombination [[2](#_ENREF_2)] was used to generate a small deletion removing most of the coding region of *shams.* Two *piggyBac* insertions, *PBac{RB}CG9996^01256^* and *PBac{RB}CG11836^e01985^* were used to induce recombination and generate candidate *w+* deletions. Candidates were confirmed by two-sided PCR using the following primer pairs (5’ to 3’): CAATGATATCTAATTCGTGAACATGAGCCTA and CCTCGATATACAGACCGATAAAAC; TCCAAGCGGCGACTGAGATG and ATGCATTTATCATGGGTTTCGCTGA. Complementation tests with a large deficiency *Df(3R)BSC494* deleting *shams* were performed at 30°C to confirm candidate deletions.

To examine the effects of loss of *shams* on the *Notch* haploinsufficient phenotypes, *Notch^55e11^*/FM7; *shams^e01256^/TM6, Tb^1^* females were crossed to *shams^e01256^/TM6, Tb* males and raised at designated temperatures. A similar crossing scheme was used to examine the effect of loss of *shams* on the gain-of-function allele, *N^Ax-E2^*. To generate MARCM clones of *shams*, *FRT82B shams^Δ34^/TM6, Tb* males were crossed to *y w Ubx-FLP tub-GAL4 UAS-GFP^nls^-6X-Myc; FRT82B y^+^ tub-GAL80/TM6, Ubx* females.

**Adult *Drosophila* Images**

For adult head images, flies were placed at -20°C overnight and then affixed to a slide. A Zeiss AxioCam MRm camera mounted on an Imager.Z1 microscope was used to obtain stacks of images from adult structures. The Z-Focus software (Bernard Instruments, Houston, TX) was used to obtain the final images. Adult wings were incubated in 100% ethanol for 2-3 minutes, dried and mounted in the DPX medium (Electron Microscopy Sciences).

**Molecular Biology**

*shams* and *CG11836* genomic regions were amplified by PCR, cloned into *attB-P[acman]-ApR* vector [[1](#_ENREF_1)] and verified by sequencing. Shams, GXYLT1, and XXYLT1 overexpression transgenes were generated by cloning the cDNAs into *pUASattB* vector [[3](#_ENREF_3)] containing an HA tag inserted 5’ of the multiple cloning site.

**Glycosyltransferase Assays.** Synthetic compounds (Xyl-Glc-R, Glc-R and *para*-nitrophenol-linked carbohydrates) were tested as acceptors at a final concentration of 100 µM. The acquired enzymatic activity was expressed as nmol xylose transferred per nmol enzyme per hour based on the protein quantification by a Coomassie Blue staining using Protein A as standard. Activity on Notch was tested on *Drosophila* Notch EGF16-20, amplified from *Drosophila* cDNA with primers GCAACTCGAGAAAGCAGATCAACGAATGCGAATC and CAAGTCTAGAGTCGTCAATATTCGTTTCGCAC and cloned via the inserted *XhoI* and *XbaI* restriction sites into *pFast-Bac1* encoding C-terminal Myc/His. Ten µl of purified EGF16-20 was incubated in the presence of 100 µM cold UDP-Xylose as donor-substrate and 10 µl bead-coupled enzyme or, as control, empty beads for 10 h at 27°C and analyzed by mass spectrometry. Protein bands were separated by SDS-PAGE, excised, and trypsin digested. Reverse phase chromatography/mass spectrometry using acetonitrile as eluent was performed on a Waters nanoACQUITY UPLC device coupled online to an Esi Q-TOF Ultima (Waters) as described [[4](#_ENREF_4)].

1. Venken KJ, He Y, Hoskins RA, Bellen HJ (2006) P[acman]: a BAC transgenic platform for targeted insertion of large DNA fragments in D. melanogaster. Science 314: 1747-1751.

2. Parks AL, Cook KR, Belvin M, Dompe NA, Fawcett R, et al. (2004) Systematic generation of high-resolution deletion coverage of the Drosophila melanogaster genome. Nat Genet 36: 288-292.

3. Bischof J, Maeda RK, Hediger M, Karch F, Basler K (2007) An optimized transgenesis system for Drosophila using germ-line-specific phiC31 integrases. Proc Natl Acad Sci U S A 104: 3312-3317.

4. Sethi MK, Buettner FF, Ashikov A, Krylov VB, Takeuchi H, et al. (2012) Molecular cloning of a xylosyltransferase that transfers the second xylose to O-glucosylated epidermal growth factor repeats of notch. J Biol Chem 287: 2739-2748.
